# Transcriptomes of the Premature and Mature Ovaries of an Ascidian, *Ciona intestinalis*

**DOI:** 10.3389/fendo.2017.00088

**Published:** 2017-04-24

**Authors:** Tsuyoshi Kawada, Akira Shiraishi, Masato Aoyama, Honoo Satake

**Affiliations:** ^1^Bioorganic Research Institute, Suntory Foundation for Life Sciences, Kyoto, Japan; ^2^Faculty of Bioscience, Nara Woman’s University, Nara, Japan

**Keywords:** ascidian, *Ciona intestinalis*, premature, ovary, transcriptome

Oogenesis and folliculogenesis are key steps in reproduction leading to preservation of species. Oocytes are generated from primordial germ cells, mature in the ovaries, and full-grown oocytes are ovulated into oviducts. In vertebrates, follicle maturation is regulated by both the hypothalamus–pituitary–gonad (HPG) axis and the HPG axis-independent process. The former is initiated after puberty and induces oocyte maturation and ovulation (1–4). In brief, gonadotropin-releasing hormone, produced in the hypothalamus, is secreted into the pituitary and induces the release of gonadotropins to the circulatory system (1, 2). Subsequently, gonadotropins trigger oocyte maturation and ovulation *via* activation of multiple pathways in granulosa and theca cells (3, 4). By contrast, the HPG axis-independent reproductive system, which mainly functions in the premature ovaries, is responsible for the growth of early-stage follicles (e.g., primordial, primary, secondary, and preantral follicles in mammals) that are not regulated by gonadotropins. To date, however, the molecular mechanisms underlying HPG axis-independent oogenesis and folliculogenesis remain largely unknown, as most studies of reproductive biology have focused on mature ovaries. Indeed, little is known about the transcriptomes of premature ovaries of vertebrates, with most data of transcriptomes and conventional expressed sequence tags (ESTs) originating from adult ovaries. Furthermore, invertebrates are not endowed with the HPG axis (no hypothalamus, pituitary, or closed circulation system), suggesting that the HPG axis may have emerged along with the acquisition of the hypothalamus, pituitary, and closed circulation system during the evolution of chordates. In other words, it is presumed that HPG axis-independent reproductive systems are conserved in vertebrates and invertebrate chordates such as ascidians.

Ascidians, or sea squirts, are marine invertebrate deuterostomes, belonging to the subphylum Tunicata or Urochordata within the phylum Chordata. Their phylogenetic position as protochordates has provided attractive and useful targets for wide-ranging biological research, including developmental biology, evolutionary biology, endocrinology, neuroendocrinology, and neuroscience. *Ciona intestinalis* is a cosmopolitan ascidian species and has outstanding advantages as a model organism (5–9). In particular, the whole-genome sequence, various ESTs, and microarray analysis data enable various gene model predictions, homology searches, and comprehensive comparisons with genomes and transcriptomes of other species^1^ (6–9). At present, approximately 17,000 ESTs of adult *Ciona* ovaries have been available, and microarray analysis comparing the *Ciona* ovary and central nervous system has detected several ovary-selective gene expressions (10). Furthermore, a *Ciona* tachykinin homolog, Ci-TK, was shown to specifically induce growth of vitellogenic oocytes by activating cathepsin D (11–13). These findings indicate that *C. intestinalis* has prominent potential as a model organism for research on HPG axis-independent folliculogenesis and the evolutionary process of folliculogenesis throughout chordates. By contrast, gene expression profiles for the *Ciona* premature ovaries have yet to be verified, which hampers investigation of the developmental process of the *Ciona* ovary. This report describes the gene expression profile of premature ovaries of *C. intestinalis*, which is expected to contribute a great deal not only to investigations of the maturation process of the *Ciona* ovary but also to the evolutionary aspect of oogenesis and folliculogenesis throughout chordates.

*Ciona intestinalis* was purchased from the Maizuru Fisheries Research Station of Kyoto University or Misaki Marine Biological Station of the University of Tokyo. The ascidians were maintained in sea water at 18°C until use. Mature and premature ovaries were dissected from 1.5-month (premature) and 4-month (mature) ascidians, and their morphology was observed using a stereoscopic microscope (M205FA: Leica, Wetzlar, Germany). The ovaries were fixed overnight in 4% paraformaldehyde at 4°C and were dehydrated and embedded in paraffin. The embedded ovaries were cut with 10-µm sections, followed by exclusion of the paraffin. The sections were soaked in hematoxylin solution for 1 min and eosin solution for 3 min. After dehydration, the sections were observed using a microscope (ECLIPSE600: Nicon, Tokyo, Japan).

Total RNA was extracted from the ovaries of ascidians using Sepasol-RNA I (Nacalai tesque, Kyoto, Japan). Each sample consists of RNA isolated from three ascidians, with two such samples obtained from premature and mature ovaries. The quality of the RNA samples was evaluated using BioAnalyzer (Agilent Technologies, Santa Clara, CA, USA) with RNA6000 Nano Chip. A 1-µg aliquot of total RNA from each sample was used to construct cDNA libraries using TruSeq Stranded mRNA Sample preparation kit (Illumina, San Diego, CA, USA), according to the manufacturer’s instructions. The resulting cDNA libraries were validated using BioAnalyzer with DNA1000 Chip and quantified using Cycleave PCR Quantification Kit (TAKARA BIO Inc., Kyoto, Japan). Single end sequencing using 101 cycles was performed using HiSeq1500 (Illumina) in the rapid mode. Total reads were extracted with CASAVA v1.8.2 (Illumina). Then, PCR duplicates, adaptor sequences, and low quality reads were removed from the extracted reads. Briefly, if the first 10 bases of the 2 reads were identical and the entire reads exhibited >90% similarity, the reads were considered PCR duplicates. The remaining reads were then aligned using Bowtie version 2.2.3 to the *C. intestinalis* genes (KH, ver. 2013), which was downloaded from Ghost Database (see text footnote 1). After two fractions of premature and mature ovaries were averaged, reads were aligned to obtain a reliable fragment per kilobase of transcript per million mapped reads (FPKM). An FPKM value >1 was considered indicative of expressed genes in ascidian ovaries. Genes satisfying the condition of FPKM^premature ovary^ value/FPKM^mature ovary^ value >1.5 were defined as those upregulated in premature ovaries, whereas genes satisfying the condition of FPKM^premature ovary^ value/FPKM^mature ovary^ <0.67 were defined as those upregulated in mature ovaries. RNA-seq for the premature and mature ovaries yielded 26 million and 15 million reads, respectively, for 101 paired-end reads. The resultant fastq files were deposited to the SRA database (biosample accession no. PRJN345247). The reads from each ovary were mapped on gene models in the *C. intestinalis* gene database.^2^ At this step, 53,968 and 54,873 transcripts were mapped from reads of premature and mature ovaries, respectively. We employed the genes with FPKM values >1, and eventually identified 6,893 and 7,357 non-redundant genes from premature and mature ovaries, respectively. Previous EST analysis, which was performed only for the mature ovary, detected 4,457 non-redundant genes (6), whereas our present RNA-seq detected more than 1.65 times the number of genes, confirming the usefulness of these data for diverse biological studies. Gene expression levels were assessed using FPKM values. Scatter plots suggested that many genes were expressed in premature and mature ovaries (Figure 1A). Homology searching by blastp on the NCBI Swissprot database under the condition of *e*-value <0.00001 showed that 97.6% (6,731) or 97.8% (7,188) of genes expressed in premature and mature ovaries, respectively, are homologous to known proteins.

**Figure 1:**
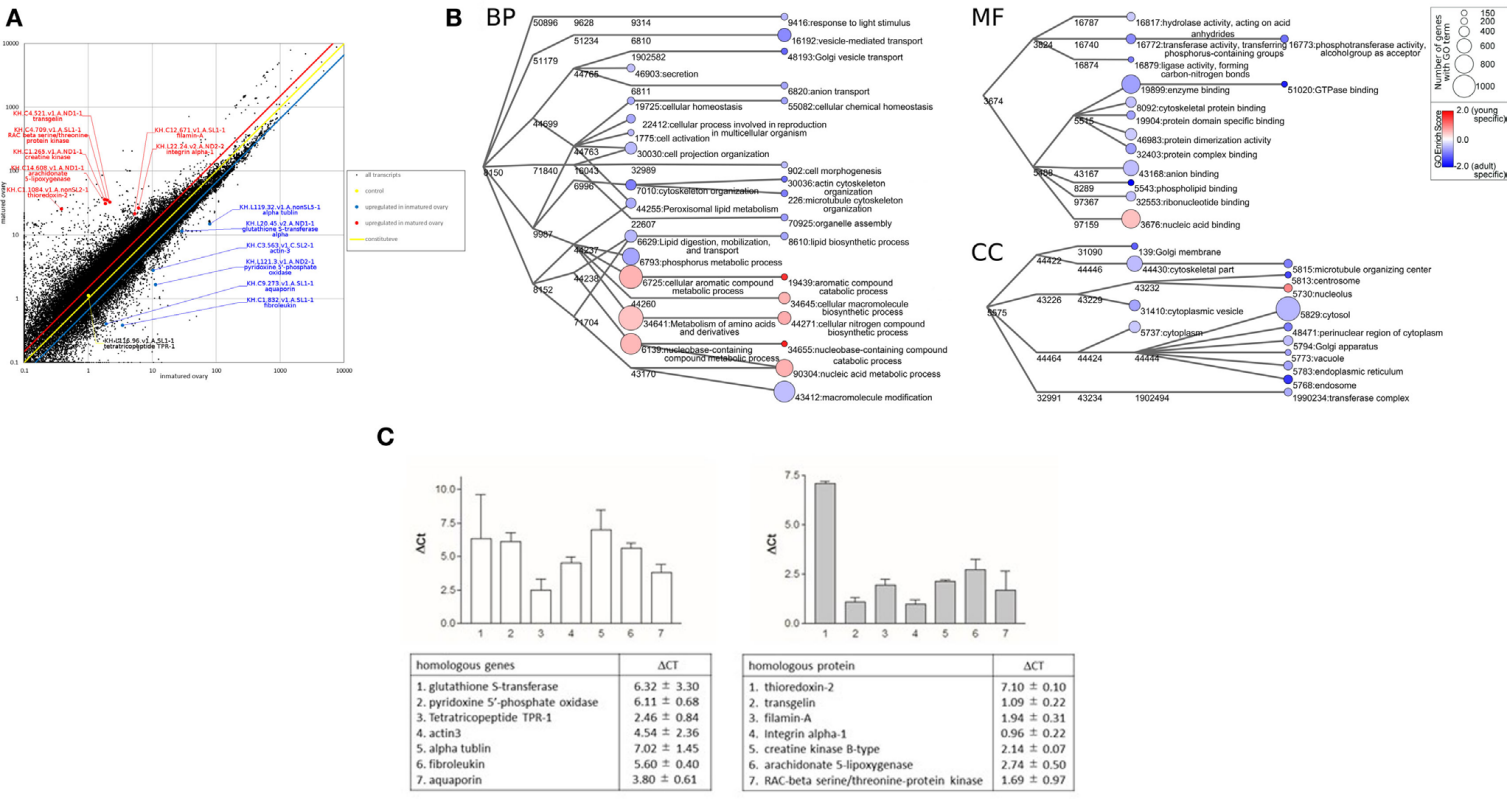
**Transcriptomic analyses of *Ciona intestinalis* ovaries**. **(A)** Comparative scatter plot analysis of gene expression levels in premature and mature ovaries. The *x*- and *y*-axes represent the expression of each gene in premature and mature ovaries, respectively. **(B)** Gene ontology (GO) enrichment analysis of differentially expressed genes in premature and mature ovaries. GO was categorized as “Biological Process” (BP), “Cellular Component” (CC), and “Molecular Function” (MF). Each node represents a GO term. The branches of GO hierarchical trees without significantly enriched GO terms are not shown. The circle size of each node indicates the number of genes with that GO term. The color of each node represents the enrichment score. Red and blue colored nodes indicate the high enrichment scores of expressed genes in premature and mature ovaries, respectively. Edges represent “is_a” connections between GO terms. **(C)** Real-time PCR analysis of several individual genes expressed in premature (left panel) and mature (right panel) ovaries. Detailed information on genes and primers is summarized in Table S1 in Supplementary Material.

Subsequently, putative genes for the transcripts were annotated based on a homology search of the Swissprot database under the condition of *e*-values <0.00001, using Blast2GO software (version 3.3) with default parameters (14). These ascidian genes were mapped and annotated using Blast2GO software with default parameters, and gene ontology (GO) terms [molecular function (MF), biological process (BP), and cellular component (CC)] were annotated for each gene. The annotated biological information was compared using GO “is_a” graphs, plotted using Cytoscape,^3^ as described previously (15, 16). To quantify the enrichment of GO terms, enrichment scores were calculated as

$$\text{Enrich }(\text{GO})=\log_{2}\frac{\frac{N_{\text{premature ovary}\ll \text{mature ovary}}(\text{GO})}{N_{\text{total}}(\text{premature ovary})\ll \text{mature ovary)}}}{\frac{N_{\text{mature ovary}\ll \text{premature ovary}}(\text{GO})}{N_{\text{total}}(\text{mature ovary})\ll \text{premature ovary)}}}$$

where *N*_X_(GO) indicates the frequency of each GO term for premature ovary (premature ovary ≪ mature ovary)- or mature ovary (mature ovary ≪ premature ovary)-specific genes (i.e., X), and *N*_total_(X) indicates the frequency of premature ovary (premature ovary ≪ mature ovary)- or mature ovary (mature ovary ≪ premature ovary)-specific genes (i.e., X) mapped to each GO term in the Blast2GO results. For collection, a pseudo-count was set at fixed value of 0.05.

Gene ontology analysis using Blast 2GO indicated that frequent GO terms were similar for premature and mature ovaries. Subsequently, we calculated FPKM^premature ovary^/FPKM^mature ovary^ ratios to identify genes upregulated in premature or mature ovaries, indicating that 1,739 genes and 5,753 genes with FPKM^premature ovary^/FPKM^mature ovary^ ratios of >1.5 and <0.67, respectively, were upregulated in premature and mature ovaries (Table S1 in Supplementary Material). Furthermore, the enrichment scores in the GO terms of these genes were calculated to examine the features of premature and mature ovaries. The annotated biological information was compared in premature and mature ovary using a heat map in graph view (Figure 1B). Nodes represent ontology terms with lower GO < 4 and edges are “is _a” relations stored in the GO database. The size and color of nodes represent the number of genes categorized by each GO term and the enrichment score of each GO term. For simple interpretation, a GO term with GO level <2 or GO term <150 genes is represented as a 0-sized node. Figure 1B shows three enrichment analyses for BP, CC, and MF which are representative GO categories. Enrichment analysis of BP indicates that genes included in “cellular aromatic compound metabolic process” (GO:0006725), “nucleobase-containing compound metabolic process” (GO:0006139), “metabolism of amino acids and their derivatives” (GO:0034641), and “cellular macromolecule biosynthetic process” (GO:0034645) were upregulated in premature ovaries. Genes in these categories participate in synthesis of nucleic acids or amino acids, suggesting that premature ovaries preferentially produce and store basal materials for ovary maturation. In mature ovaries, genes included in “actin cytoskeleton organization” (GO:0030036), “microtubule cytoskeleton organization” (GO:0000226), “Golgi vesicle transport” (GO:0048193), “vesicle-mediated transport” (GO:0016192), “anion transport” (GO:0006820), “response to light stimuli” (GO:0048193), “cellular chemical homeostasis” (GO:0055082), “cell morphogenesis” (GO:0000902), “organelle assembly” (GO:0070925), “lipid biosynthesis” (GO:0006610), and “macromolecule modification” (GO:0043412) were upregulated. These genes are responsible for various biological processes, differing from those in premature ovaries. Enrichment analysis of CC showed that genes included in “nucleolus” (GO:0005730) and “nucleic acid binding” (GO:0003676) were upregulated in premature ovaries. By contrast, enrichment analyses of CC indicated that genes related to various organelles were upregulated in mature ovaries: “centrosome” (GO:0005813), “endoplasmic reticulum” (GO:0005783), “Golgi membrane” (GO:0000139), “Golgi apparatus” (GO:0005794), “endosome” (GO:0005768), “vacuole” (GO:0005773), “cytoplasm” (GO:0005737), “cytoplasmic vesicle” (GO:0031410), “perinuclear region of cytoplasm” (GO:0048471), and “cytosol” (GO:0005829). Likewise, enrichment analysis of MF indicated that genes related to various enzyme activities and molecular bindings were expressed in mature ovaries: “hydrolase activity” (GO:0016817), “transferase activity” (GO:0016772), “ligase activity” (GO:0016879), “enzyme binding” (GO:0019899), “cytoskeletal protein binding” (GO:0008092), “protein domain specific binding” (GO:0019904), “protein dimerization activity” (GO:0046983), “protein complex binding” (GO:0032403), “anion binding” (GO:0043168), “phospholipid binding” (GO:0005543), and “ribonucleotide binding” (GO:0032553). These expression profiles suggest higher and more extensive cellular activity in mature ovaries than in premature ones, ultimately leading to various biological functions including oocyte growth, maturation, ovulation, and transport of oocytes to the oviduct. Moreover, GO analysis of genes included in “reproduction” (GO:0000003), which are also included in BP, CC, or MF, indicated that various genes related to translation, such as ribosomal proteins, were upregulated in premature ovaries (Table S1 in Supplementary Material). This result also supports a notion that the synthesis of the large numbers of proteins was initiated in premature ovaries. By contrast, genes encoding transcriptional factors, signaling molecules, and cytoskeleton proteins were shown to be upregulated in mature ovaries (Table S1 in Supplementary Material).

Subsequently, real-time PCR confirmed the differential expression of several of these genes in premature or mature ovaries (Figure 1C). Real-time PCR was performed using a CFX96 Real-time System and SsoAdvanced™ Universal SYBR Green Supermix (Bio-Rad laboratories, Hercules, CA, USA). The total volume of each real-time PCR reaction mixture was 10 µl and contained 10-ng template cDNA, 250 nM of each primer, and 5 µl SYBR Green Master Mix solution. The real-time PCR program consisted of denaturation at 95°C for 30 s, followed by 44 cycles of denaturation at 95°C for 15 s, and annealing and extension at 60°C for 30 s. Melting curve analysis of the amplified PCR products was performed to confirm the absence of primer dimers. Gene expression levels were evaluated using the Δ*C*_t_ method. *C*_t_ value represents the PCR cycle number when the PCR product reached the detectable level, and Δ*C*_t_ represents the difference between *C*_t_ values of PCR products prepared from premature and mature ovaries. The primers used for real-time PCR were designed using Primer3Plus (Table S1 in Supplementary Material) and obtained from Thermo Fisher Scientific.

A homologous gene to alpha-tubulin, categorized as “cytoskeletal part” (GO:0044430), showed 130-fold higher expression in premature ovaries, compared to mature ovaries (Figure 1C). This result is in good agreement with a recent report that the mutation in human tubulin gene abolishes the microtubule formation and arrested spindle formation of oocytes (17), thereby suggesting that the tubulin-associated spindle formation occurs in early-stage oocytes in premature ovaries of infant *C. intestinalis*.

The expression level of another cytoskeleton-related gene, a cytoplasmic actin gene expressed in unfertilized eggs (18), was approximately 23-fold higher in premature ovaries than in mature ovaries (Figure 1C). In addition, the gene encoding actin-binding protein, filamin-A, was approximately fourfold upregulated in mature ovaries (Figure 1C). Filamin-A plays important roles in cell adhesion and migration (19, 20), supporting a view that upregulation of filamin-A gene is involved in organization of the cortical region of oocytes, nuclear migration in oocytes, and cell elongation in mature follicles.

As stated above, enrichment analysis indicated that genes categorized as “metabolism of amino acids and derivatives” (GO:0034641) were upregulated in premature ovaries. As depicted in Figure 1C, real-time PCR showed that two of these genes, pyridoxine 5′-phosphate oxidase and glutathione *S*-transferase, were upregulated 69- and 80-fold, respectively, in premature ovaries. Pyridoxine 5′-phosphate oxidase is responsible for the production of amino acids and sphingolipid *via* synthesis of vitamin B6 (21, 22). Glutathione *S*-transferase alpha acts as an antioxidant in cytosol and participates in cysteine synthesis (23). These proteins are highly likely to supply basal materials for cellular components such as amino acids, leading to maturation of oocytes and ovaries. In addition, genes encoding a tetratricopeptide, a fibroleukin, and an aqua-glyceroporin were upregulated approximately 6-, 49-, and 14-fold, respectively, in premature ovaries (Figure 1C). A teleost-specific aquaporin, AQP1ab, was found to be expressed in mature oocytes and involved in oocyte hydration for formation of floating eggs (24). Aqua-glyceroporin is involved in the absorption of glycerol and other solutes as well as of water (25), suggesting that these solutes, incorporated by aqua-glyceroporin, play a physiological role in *Ciona* premature ovaries. Altogether, this is the first report showing that an aquaporin gene was expressed in premature ovaries in any organism.

Real-time PCR also showed that three genes related to cell proliferation, genes encoding a RAC-beta serine/threonine-protein kinase, an integrin alpha-1, and a creatine kinase were upregulated threefold, twofold, and fourfold, respectively, in mature ovaries. RAC-beta serine/threonine-protein kinase is a member of the PI3K/Akt family that is implicated with various signaling cascades (26). Integrin alpha-1 also has multiple biological roles in cell adhesion (27), and creatine kinase is involved in basal ATP metabolism to acquire energy used for cell proliferation (28). Thioredoxin-2 is a protein localized to the mitochondria and reduces oxygen radicals generated from electron transport chains (29), suggesting that approximately 137-fold upregulation of thioredoxin-2 gene is functionally correlated with biological roles of mitochondria in generating energy. Moreover, a transgelin gene categorized as “cytoskeletal part” (GO:0044430) and a filamin-A gene categorized as “cytoskeletal protein binding” (GO:0008092) were upregulated approximately twofold and fourfold, respectively, in mature ovaries (Figure 1C). Both proteins are involved in actin binding in mature ovaries, for example, during cell adhesion or migration (30). In addition, an arachidonate 5-lipoxygenase gene categorized as “lipid biosynthetic process” (GO:0008610) was approximately upregulated sevenfold in mature ovaries (Figure 1C). Collectively, these expression profiles support a view that biological pathways for acquiring energy and the production of various cellular components including cytoskeltons were activated in mature ovaries, leading to the induction of oocyte maturation, ovulation, and the transport of oocytes into the oviduct.

This study reports fundamental transcriptome data for premature and mature ovaries of an ascidian, *C. intestinalis*. This is the first report describing transcriptomes of premature ovaries from a deuterostome invertebrate. Consequently, the present data pave the way for investigating the molecular mechanisms underlying oogenesis and folliculogenesis in infant organisms and the evolutionary process of follicle maturation in chordates.

## Author Contributions

HS designed and supervised experiments. TK, AS, MA, and HS performed experiments, prepared figures, interpreted data, and wrote the manuscript.

## Conflict of Interest Statement

The authors declare that the research was conducted in the absence of any commercial or financial relationships that could be construed as a potential conflict of interest.

## Footnotes

^1^http://ghost.zool.kyoto-u.ac.jp/download_kh.html.

^2^http://ghost.zool.kyoto-u.ac.jp/cgi-bin/gb2/gbrowse/kh/.

^3^http://www.cytoscape.org/.

## Supplementary Material

Table S1 **List of reproduction-related genes upregulated in premature and mature ovaries, along with their primer sequences for real-time PCR**.
